# Effects of buffer composition and plasmid toxicity on electroporation-based non-viral gene delivery in mammalian cells using bursts of nanosecond and microsecond pulses

**DOI:** 10.3389/fbioe.2024.1430637

**Published:** 2024-07-10

**Authors:** Eivina Radzevičiūtė-Valčiukė, Jovita Gečaitė, Austėja Balevičiūtė, Anna Szewczyk, Augustinas Želvys, Barbora Lekešytė, Veronika Malyško-Ptašinskė, Eglė Mickevičiūtė, Paulina Malakauskaitė, Julita Kulbacka, Vitalij Novickij

**Affiliations:** ^1^ State Research Institute Centre for Innovative Medicine, Department of Immunology and Bioelectrochemistry, Vilnius, Lithuania; ^2^ Faculty of Electronics, Vilnius Gediminas Technical University, Vilnius, Lithuania; ^3^ Faculty of Pharmacy, Department of Molecular and Cellular Biology, Wroclaw Medical University, Wroclaw, Poland

**Keywords:** transfection, electroporation, GFP, luminescence, *in vitro*, *in vivo*, 4T1

## Abstract

Gene electrotransfer (GET) is non-viral gene delivery technique, also known as electroporation-mediated gene delivery or electrotransfection. GET is a method used to introduce foreign genetic material (such as DNA or RNA) into cells by applying external pulsed electric fields (PEFs) to create temporary pores in the cell membrane. This study was undertaken to examine the impact of buffer composition on the efficiency of GET in mammalian cells Also, we specifically compared the effectiveness of high-frequency nanosecond (ns) pulses with standard microsecond (µs) pulses. For the assessment of cell transfection efficiency and viability, flow cytometric analysis, luminescent assays, and measurements of metabolic activity were conducted. The efficiency of electrotransfection was evaluated using two different proteins encoding plasmids (pEGFP-N1 and Luciferase-pcDNA3). The investigation revealed that the composition of the electroporation buffer significantly influences the efficacy of GET in CHO-K1 cell line. The different susceptibility of cell lines to the electric field and the plasmid cytotoxicity were reported. It was also shown that electroporation with nanosecond duration PEF protocols ensured equivalent or even better transfection efficiency than standard µsPEF. Additionally, we successfully performed long-term transfection of the murine 4T1 cell line using high-frequency nanosecond PEFs and confirmed its’ applicability in an *in vivo* model. The findings from the study can be applied to optimize electrotransfection conditions.

## 1 Introduction

Electroporation (EP) is a physical phenomenon in which cell membranes polarize due to an external electric field and nanoscale aqueous pores in a phospholipid bilayer are formed. This process facilitates the delivery of usually impermeable molecules into the cell (Kotnik et al., 2019; Tylewicz, 2020). EP is widely used as a non-viral gene delivery technique, termed gene electrotransfer (GET). This physical method improves cellular uptake of DNA, plasmid DNA (pDNA), and RNA and can be applied to both in *vitro* and *in vivo* models (Lambricht et al., 2015; Cervia and Yuan, 2018; Rakoczy et al., 2022). The wide applicability of GET involves treatment of cancer and other diseases (Li and Huang, 2000; Fewell et al., 2005) and DNA vaccination (Chattergoon et al., 1997). Electroporation presents numerous advantages in comparison to viral gene transfer methodology, including the ability to repeatedly administer to tissues without triggering substantial immune responses or toxicity, minimizing the occurrence of transgene expression at non-target sites, good stability, the capability to carry large amounts of material, high therapeutic efficacy, and low cost. While the standard viral gene transfer technique is limited by significant health risk associated with viral components in vectors cytotoxicity, immunogenicity and the potential for mutagenesis through chromosomal integration (Henshaw and Yuan, 2012; Li et al., 2012; Karagöz et al., 2018; Kumar et al., 2021). Nevertheless, the electroporation-based gene transfer methodology also faces limitations. These include relatively low transfection efficiency compared to viral delivery techniques, as well as compromised cell viability following treatment, particularly when employing high-energy electric pulses (Henshaw and Yuan, 2012). Therefore, there is a significant emphasis on the importance of research in electroporation-based gene electrotransfer, particularly in parametric optimization to ensure higher transfection efficacy.

In addition, it is important to consider that GET is a multistep process that includes electropermeabilization of the cell membrane lipid bilayer, electrophoretic migration of DNA toward the membrane with subsequent interaction, translocation across the membrane, migration toward the nucleus, and passage through it to ensure gene expression (Gong et al., 2022; Rakoczy et al., 2022), where the interaction of a DNA molecule with the cell membrane was marked as one of the key steps (Neumann et al., 1996; Golzio et al., 2002). Moreover, maintaining high viability and ensuring adequate plasmid quality are imperative for successful cell electrotransfection.

It is widely acknowledged that primary factors influencing the effectiveness of electroporation include electric field strength, pulse number, pulse duration, and applied frequency. Additionally, the efficiency can be influenced by temperature, plasmid quality, molecular size, and concentration as well as properties of the targeted cells (shape, cell membrane charge, cell density) (Golzio et al., 1998; Čegovnik and Novaković, 2004; Lesueur et al., 2016; Desai et al., 2017; Chopra et al., 2020; Sherba et al., 2020; Radzevičiūtė et al., 2022a). Studies have additionally shown that the characteristics of the electroporation buffer, including conductivity, osmolarity, and ionic composition, used during and post-electroporation, greatly influence gene electrotransfer efficiency (Neumann et al., 1996; Hristova et al., 1997; Flanagan et al., 2011; Haberl et al., 2013; Chopra et al., 2020).

In the matter of buffer ionic composition, it is noted that divalent cations such as Ca^2+^ and Mg^2+^ are necessary for the formation of DNA–membrane complex during the PEFs application. They act as a bridge between negatively-charged DNA and the negatively-charged cell plasma membrane and thus improve DNA–membrane binding (Wong and Neumann, 1982; Haberl et al., 2013). Other study results revealed that increased concentration of Mg^2+^ ions in electroporation buffer results in stronger interaction of DNA with the membrane and higher cell viability post-treatment. However, lower gene electrotransfer efficiency was reported (Haberl et al., 2010). Furthermore, the presence of Mg^2+^ in electroporation buffers may enhance cell viability by accelerating the restoration of ion homeostasis, even with higher energy pulses used (Sherba et al., 2020). The lower electrotransfection efficiency when higher concentration of Mg^2+^ ions is used can be explained by the increased activity of DNAse enzyme (Tullis and Price, 1974) or it can be hypothesized that Mg^2+^ ions bind DNA to the cell surface with such strength that it cannot pass into the cell during EP (Haberl et al., 2010). The role of Ca^2+^ ions in the EP buffer has also been reported. Data from *in vivo* studies demonstrated that the peak of transgene expression was achieved when Ca^2+^ levels were optimized (20–100 mmol/L) (Suzuki et al., 2003). Contrastingly, another group reported that the presence of Ca^2+^ ions in the DNA solution significantly inhibited transgene expression following EP procedure (Zhao et al., 2006). The inhibitory effect of Ca^2+^ ions may be associated with the properties of the cell membrane following electroporation and the subsequent resealing process (Ciobanu et al., 2018; Shi et al., 2022; Li and Shaw, 2023). Thus, to achieve high efficiency mammalian cell transfection additional optimization experiments need to be performed.

Most electric pulse protocols utilized for gene electrotransfer typically involve the application of long-duration electrical pulses (micro-millisecond range) (Potter and Heller, 2003; Cemazar et al., 2009; Kandušer et al., 2009; Nomura et al., 2016) or treatment modalities like European Standard Operating Procedures for Electrochemotherapy (ESOPE) (Cukjati et al., 2007; Miklavčič et al., 2012). Experimental data demonstrates that even longer than microsecond duration pulses of higher amplitude ensured better gene electrotransfer and longer protein expression in *vivo* model (Zampaglione et al., 2005; Prud’homme et al., 2006; Mir, 2009). This can be attributed to the increased electrophoretic effect observed when longer duration electric pulses are applied (Sukharev et al., 1992). However, it is noted that longer-duration pulses cause undesirable muscle contractions (Golberg and Rubinsky, 2012) or electrochemical reactions (Rodaite-Riseviciene et al., 2014), which could potentially impact the quality of plasmid DNA and consequently have a negative effect on GET efficiency (Cukjati et al., 2007; Arkhangelsky et al., 2008; Zhao et al., 2021). One potential method to mitigate these adverse effects is through the application of shorter pulsed electric fields in the nanosecond range (nsPEFs). Recent studies revealed that pre-treatment with nsPEF before msPEF increased transfected cells viability and gene expression (Guo et al., 2014). Additionally, we presented a proof of concept (Ruzgys et al., 2018) and published experimental data that high-frequency sub-microsecond PEF protocols can ensure high transfection efficiency and cell viability (Novickij et al., 2020; Radzevičiūtė et al., 2022b). Further advantages of applying high-frequency nsPEFs include the reduction in the generation of reactive oxygen species (ROS) (Radzevičiūtė-Valčiukė et al., 2023). The absence of an electrophoretic component in the pulse itself is compensated by the accumulation of residual transmembrane potential (TMP) during the bursts, leaving the cells polarized. This polarization may enhance the stability and/or size of the pores (Radzevičiūtė et al., 2022a). This is a relatively new (first works occurring in 2018) and evolving field that necessitates more applied research to further substantiate the feasibility of nsPEF in GET. It is also necessary to study how electroporation buffer composition as well as different EP parametric protocols affects GET efficacy.

Therefore, in this study, we focused on the effects of buffer composition on GET efficiency in mammalian cells using high-frequency nanosecond (ns) in comparison to standard microsecond (µs) pulses. The main goal of this study was to define electroporation buffer composition and develop long-term transfected murine (4T1) cancer cell line, as a proof of concept.

## 2 Materials and methods

### 2.1 Electroporation setup and parameters

Electroctroporation experiments were performed using a 3 kV, 100 ns–1 m square-wave pulse generator (VilniusTECH, Vilnius, Lithuania) (Novickij et al., 2016) and a commercially available electroporation cuvette with a 1 mm gap between electrodes (Biorad, Hercules, United States). The voltage applied to the cuvette varied according to selected protocols. For evaluation of electrotransfection (pEGFP-N1 plasmid) efficacy dependency on EP buffer (1–8) and cell viability, we have conducted tests with ESOPE-like microsecond (µsPEF) and nanosecond (nsPEF) pulsing protocols. The µsPEF protocol involved pulses of 100 µs duration and electric field strength values of 1.2 kV/cm and 1.5 kV/cm, using four and eight pulses at a pulse repetition frequency of 1 Hz. For nsPEF, we applied amplitudes of 4 kV/cm and 5 kV/cm with 500 and 250 pulses, respectively, at a repetition frequency of 1 MHz. Permeabilization assay was performed for both µsPEF and nsPEF using 1.2 kV/cm and 2.5–10 kV/cm pulse amplitudes, respectively. Finally, only two electric protocols [µsPEF (1.2 kV/cm × 100 µs × 8, 1 Hz) and nsPEF (2.5–10 kV/cm × 300 ns × 250, 1 MHz)], were used in electrotransfection with Luciferase-pcDNA3 plasmid.

### 2.2 Cell lines

Murine 4T1 (ATCC-CRL-2539), a mammary gland tumor cell line of BALB/c origin and Chinese Hamster Ovary CHO-K1 (ATCC-CCL-61) cell lines were grown in RPMI 1640 medium supplemented with glutamine, 100 U/mL of penicillin, 100 mg/mL of streptomycin, and 10% of fetal bovine serum (FBS). Cultivation of cells was carried out at 37°C with a 5% CO_2_ atmosphere. The cell culture reagents were obtained from Gibco, Thermo Fisher Scientific, Waltham, MA, United States.

The experimental day the cells were detached from the cell cultivation dish using Trypsin-EDTA solution (Thermo Fisher Scientific, Waltham, MA, United States) incubating for 3–10 min, centrifuged, and resuspended in the electroporation buffer (Table 1). For the gene electrotransfer experiments, a concentration of 6 × 10^6^ cells/mL was used. The cell membrane permeabilization and viability assays were performed at a concentration of 2 × 10^6^ cells/mL.

**TABLE 1:** The composition of used electroporation buffers.

EP buffer No.	Chemical composition
1	Sucrose 242 mM, Na_2_HPO_4_ 5.5 mM, NaH_2_PO_4_ 3 mM, MgCl_2_ 1.7 mM, pH 7.1 Chopra et al. (2019)
2	NaCl 140 mM, KCl 5.4 mM, MgCl_2_ 1.5 mM, CaCl_2_ 2 mM, glucose 10 mM, HEPES 10 mM, pH 7.3 Pakhomov et al. (2014)
3	KCl 5mM, MgCl_2_ 15 mM, HEPES 15 mM, Na_2_HPO_4_/ NaH_2_PO_4_ 150mM, mannitol 50 mM, pH 7.2 Parreno et al. (2015)
4	HEPES 21 mM, NaCl 137 mM, KCl 5 mM, Na_2_HPO_4_⋅7H_2_O 0.7 mM, dextrose 6 mM, pH 7.2 Zhou et al. (2016)
5	HEPES 10 mM, sucrose 250 mM, MgCl_2_ 1 mM, pH 7.2 Gibot et al. (2020)
6	KCl 5 mM, MgCl_2_ 15 mM, Na_2_HPO_4_/NaH_2_PO_4_ 120 mM, mannitol 50 mM, pH 7.2 Parreno et al. (2015)
7	KCl 5 mM, MgCl_2_ 15 mM, NaCl 90 mM, glucose 10 mM, Ca(NO_3_)_2_ 0.4 mM, Na_2_HPO_4_/NaH_2_PO_4_ 40 mM, pH 7.2 Parreno et al. (2015)
8	KCl 5 mM, MgCl_2_ 15 mM, NaCl 90 mM, glucose 10 mM, Ca(NO_3_)_2_ 0,4 mM, HEPES 20 Mm, Tris/HCl 75 mM, pH 7.2 Parreno et al. (2015)

Various electroporation buffers have been selected based on a review of various available electroporation-associated works involving gene transfer (Table 1). The buffers include standard sucrose and HEPES-based buffers for electroporation research (e.g., No. 1, 2, and 5) and commercially recommended buffers (e.g., No. 4), also less popular buffers from other studies focused on nucleofection are included to enable better repeatability and consolidation of knowledge (e.g., No. 3, 6–8) (Pakhomov et al., 2014; Parreno et al., 2015; Zhou et al., 2016; Chopra et al., 2019; Gibot et al., 2020)

### 2.3 Cell membrane permeabilization and detection

Cell membrane permeabilization post-electroporation was assessed with BD Accuri C6 flow cytometer (BD Biosciences, San Jose, CA, United States) using fluorescent stain Yo-Pro1 (YP1; Thermo Fisher Scientific, Waltham, MA, United States). The cells in the electroporation buffer were mixed with YP1 stain for the final stain concentration of 1 μM. The 50 μL of mixed solution were positioned between the electrodes and subjected to various EP protocols. Subsequently, they were incubated at room temperature for 3 min. Following this, 150 μL of 0.9% NaCl solution was added, and the samples were analyzed using flow cytometry. YP1 fluorescence (491⁄509) was detected using Channel FL1 (533/30 nm BPF). The results were analyzed using FlowJo software (BD, Becton Drive Franklin Lakes, NJ, United States).

### 2.4 Viability determination

After the EP treatment the cells were transferred into a flat-bottom 96-well plate, incubated for 10 min and growth media to the final 200 μL volume in each well was added. Additionally, untreated cells were used as a control for data normalization. After 24 h cell viability was assessed using PrestoBlue cell viability reagent (Thermo Fisher Scientific, Waltham, MA, United States). First, all the wells were gently washed twice with phosphate-buffered saline (PBS). Afterward, 90 µL of PBS and 10 µL of cell viability reagent were added to each well, followed by 2 h incubation. The fluorescence (Ex. 540/20 nm; Em. 620/40 nm) was measured using a Synergy two microplate reader and Gen5 software (BioTek, Shoreline, WA, United States).

### 2.5 Plasmids

Plasmids construct pEGFP-N1 (4,733 bp) carrying the gene of GFP and Luciferase-pcDNA3 [7,040 bp; Addgene plasmid #18964, a kind gift from William Kaelin, Harvard Medical School, Boston, MA, United States (Safran et al., 2006)] encoding luciferase were used. Plasmids were prepared from transfected *E. Coli* using the Plasmid Plus Giga Kit (Qiagen, Hilden, Germany), according to the manufacturer’s instructions and diluted in sterile H_2_O to a 2 mg/mL concentration.

For the long-term transfection experiments, the luciferase-pcDNA3 plasmid was restricted with Bgl II enzyme. The digestion mix with plasmid DNA was incubated for 4 h at 37°C. Afterward, the linearized plasmid was concentrated by EtOH precipitation: 0.2 volumes of CH_3_COONa (3 M, pH 4.8) and three volumes of 96% EtOH were added. Following a 20-min incubation on ice, the vial containing the precipitated DNA was centrifugation (31,840 × g for 15 min). Subsequently, the plasmid was subjected to a wash with 70% ethanol and centrifuged once more. Finally, the resulting pellet was resuspended in sterile water.

After 20 min of incubation on ice, the vial with precipitated DNA was centrifuged (31,840 × g, 15 min). Afterward, the plasmid was washed with 70% EtOH and centrifuged again. The pellet was resuspended in sterile H_2_O. The linearized plasmid (0.5 μg/μL) were used for long-term transfection.

### 2.6 Gene electrotransfer

Each electrotransfection sample was performed by mixing 30 μL of ice-cold cell suspension and 4 μL corresponding plasmid DNA. Right after electroporation, the cells were moved to a 48-well plate and kept on ice for 10 min. Subsequently, 0.5 mL of cell culture growth media was added, and cells were left for the 24 h incubation period.

**FIGURE 1:**
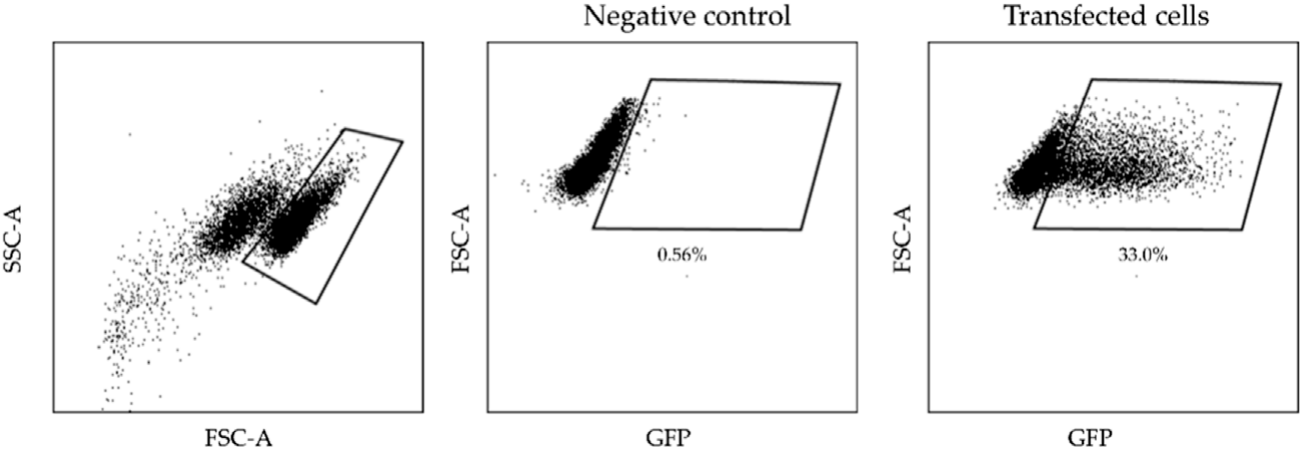
The gating strategy illustrates transfected cells analysis.

Next day cells electrotransfected with pEGFP-N1 plasmid were detached using Trypsin-EDTA solution, centrifuged (400 × g for 5 min), resuspended with 70 μL PBS and further assessed by flow cytometry (BD Accuri C6). The transfected cells fluorescence of GFP (Ex. 491⁄509) was detected using Channel FL1 (533/30 nm BPF). A shift of fluorescence spectra and the cells in the defined gate (which was defined based on the untreated, negative control) have been interpreted as fluorescence positive (transfected), while the cells outside the gate have been interpreted as non-fluorescent (nontransfected). The gating strategy for electrotransfection analysis is presented in Figure 1. The results were analysed using FlowJo software (BD, Becton Drive Franklin Lakes, NJ, United States).

The luciferase-pcDNA3 plasmid transfected cells were detached using Trypsin-EDTA solution, centrifuged (400 × g for 5 min), resuspended with 90 μL growth media and transferred into the white 96-well plates. D-Luciferin (Promega, Madison, WI, United States) was added to the cells at a final concentration of 150 μg/mL. The luminescence of luciferase-pcDNA3 plasmid expressing firefly luciferase was evaluated using a Synergy two microplate reader and Gen5 software (BioTek, Shoreline, WA, United States).

### 2.7 Long-term transfection

The 4T1 cells were electrotransfected with linearized luciferase-pcDNA3 plasmid as described above. However, after electroporation 30 μL of the sample was transferred to a Petri dish where the cells were incubated for 10 min. After incubation, 12 mL of the cell culture medium was added, and a 48-h incubation period followed. G418 Sulphate (200 μg/mL; Carl Roth GmbH, Karlsruhe, Germany) was used to select transfected 4T1 cells. Only the cells resistant to G418 remained viable and were subsequently cloned by transferring them into individual 96-well plates. Then, clones were named and grown up to a week. The optimal luminescent cell clones were chosen by comparing the maximum luminescence (in RLU) observed across the clone’s complete kinetic readings. Long-term transfected cells expressing luciferase were cultured and preserved by freezing them in a medium consisting of 10% DMSO and 90% FBS. These cells were then stored in liquid nitrogen until needed. The luciferase-expressing cell line that was established was named 4T1-Luc.

### 2.8 *In Vivo* bioluminescence assay

Female Balb/c 6–8-week-old mice were bred and housed in the mouse facility of the State Research Institute Centre for Innovative Medicine (Vilnius, Lithuania). 2 × 10^6^ 4T1-Luc cells were injected subcutaneously (s.c.) on the left flank to establish tumors.

The luminescence of established tumors was imaged by IVIS Spectrum and Living Image Software (Caliper/Perkin Elmer, Akron, OH, United States) when tumor volume reached 50 mm^3^. First, mice intraperitoneally received 15 mg/mL D-luciferin (Promega, Madison, WI, United States) solution (150 µL/mouse) and after 10–15 min luminescence intensity was visualized. The bioluminescence is proportional to the number of live, growing 4T1-Luc cells. Images were taken when the animal was under anesthesia (3% isoflurane and oxygen gas mixture).

The consent to perform animal experiments was obtained from the State Food and Veterinary Service (approval no. G2-266), carrying out the study strictly according to the Guide for the Care and Use of Laboratory Animals.

### 2.9 Statistical analysis

One-way analysis of variance (ANOVA) was used to compare different treatments. Tukey’s HSD multiple comparison test for the evaluation of the difference was used when ANOVA indicated a statistically significant result (*p* < 0.05 was considered as statistically significant). The data were post-processed using the OriginPro software program (OriginLab Corporation, Northampton, MA, United States). Each experimental point was obtained from at least three independent experiments, and results are represented as mean ± standard deviation.

## 3 Results

### 3.1 The influence of electroporation buffer composition on GET using CHO-K1 cell line

**FIGURE 2:**
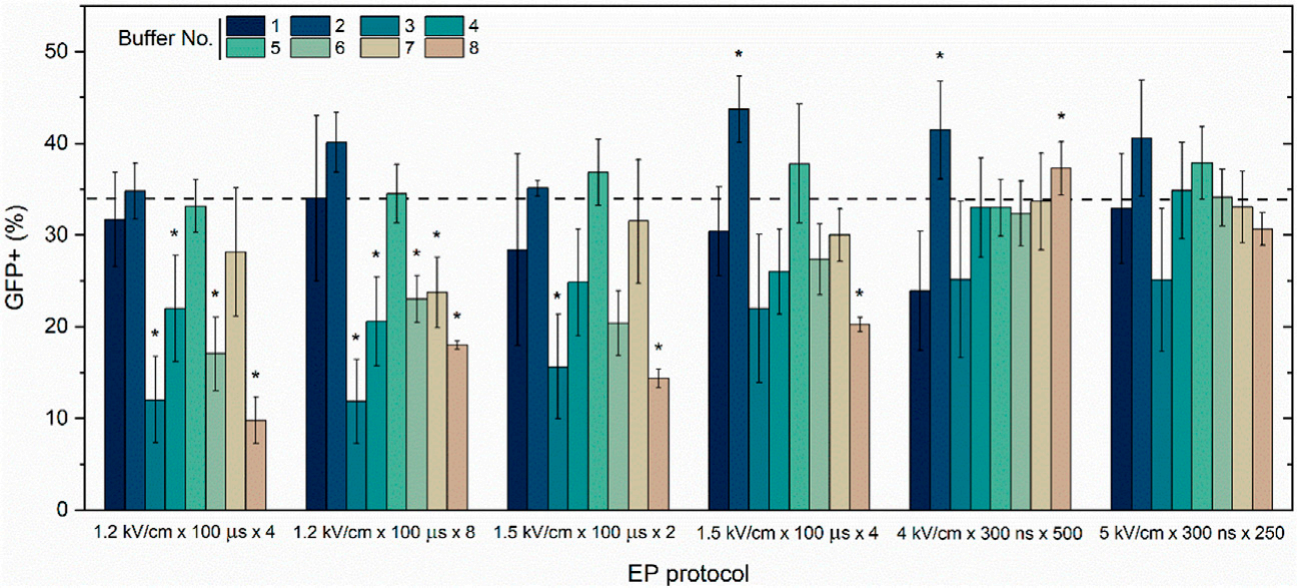
CHO-K1 cell line electrotransfection efficiency dependence on EP buffers composition (No. 1–8) and applied protocols with pEGFP-N1 plasmid encoding green fluorescent protein (GFP). Dashed line indicates average transfection efficacy using 1.2 kV/cm × 100 μs × 8 protocol and electroporation buffer No.1, which serves as a positive control. Asterisk (*) corresponds to statistically significant differences (*p* < 0.05) *versus* positive control.

Previously, as a proof of concept we have determined that 300–700 ns duration electric pulses can be used for efficient transfection (Novickij et al., 2020), therefore, we have selected optimal ns protocols (4 kV/cm × 300 ns × 500) and (5 kV/cm × 300 ns × 250) delivered at 1 MHz and compared the efficiency to microsecond procedures (100 µs). The influence of eight distinct buffer solutions (refer to Table 1) on gene electrotransfer efficiency in the CHO-K1 cell line, utilizing the pEGFP-N1 plasmid, is illustrated in Figure 2.

The CHO-K1 cell line and the standard pEGFP-N1 plasmid were selected as well-described models to ensure data repeatability and the consolidation of knowledge. The transfection efficiency is highly dependent on buffer composition, while buffer (1, 2, and 5) are the most suitable for microsecond pulses. Interestingly, the influence of buffers on transfection efficiency is significantly diminished in case of nanosecond protocols. Both ns protocols result in comparable efficiency of electrotrasfection independently on the used buffer, however, there is a clear tendency that buffer No. Three is the least suitable for GET *in vitro*.

**FIGURE 3:**
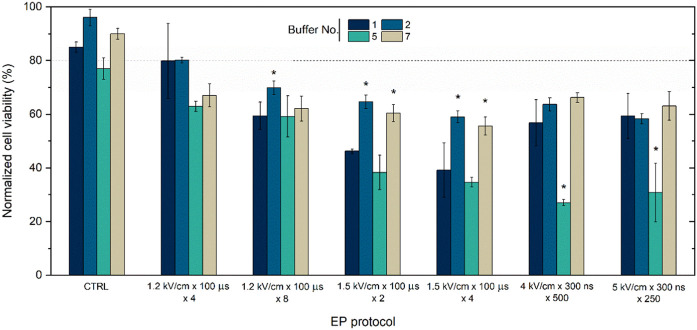
CHO-K1 cells viability dependence on EP buffers composition (No. 1, 2, 5, and 7) and applied protocols with pEGFP-N1 plasmid encoding green fluorescent protein (GFP). Dot line indicates transfection efficacy using 1.2 kV/cm × 100 μs × 8 protocol. CTRL refers to cells with plasmid in different buffer without electroporation treatment. Asterisk (*) corresponds to statistically significant differences (*p* < 0.05).

While successful plasmid DNA entry into the cell and protein production are important for transfection, viability is another crucial parameter characterizing treatment efficiency. Therefore, the study was further limited to the most suitable buffers (i.e., 1, 2, 5, and 7) and the toxicity of the treatment (PEF + toxicity of the plasmid) was evaluated. The results are summarized in Figure 3.

It can be seen (Figure 3) that the buffers are not toxic by themselves, however, when combined with electric field the overall toxicity of the treatment starts to rise and is also are highly dependent on buffer composition. Primarily, buffers numbered 1, 2, and seven reliably maintain the highest cellular viability levels, averaging over 60%. Conversely, buffer No. Five exhibits a negative impact on cell viability during GET, potentially decreasing to approximately 30%. The conventional electroporation solution, characterized by a sucrose-based composition (buffer No. 1), yields outcomes akin to buffers No. Two and seven when subjected to a voltage of 1.2 kV/cm or to nanosecond protocols. However, when exposed to increased amplitudes, such as 1.5 kV/cm with 100 µs pulses, a notable reduction in viability can be seen (Figure 3).

Usually, the GFP plasmid and CHO-K1 cell line serve as a model to predict electrotransfection efficiency and further to be used as protocol with other cell lines for long-term transfection. However, various cell lines exhibit different susceptibility to PEF and certain cell lines present challenges in achieving long-term transfected clones.

Therefore, to determine if the same tendency occurs with other cell lines and plasmids, the research was limited to marginal cases (i.e., buffers No. 1, 2, and 5), and long-term transfection with the 4T1 cell line was performed to develop a luminescent 4T1 cell line clone. However, firstly the efficiency of permeabilization to (YP) following PEF was characterized and compared with CHO-K1 cell line.

### 3.2 The influence of electroporation buffer composition on GET using 4T1 cell line

**FIGURE 4:**
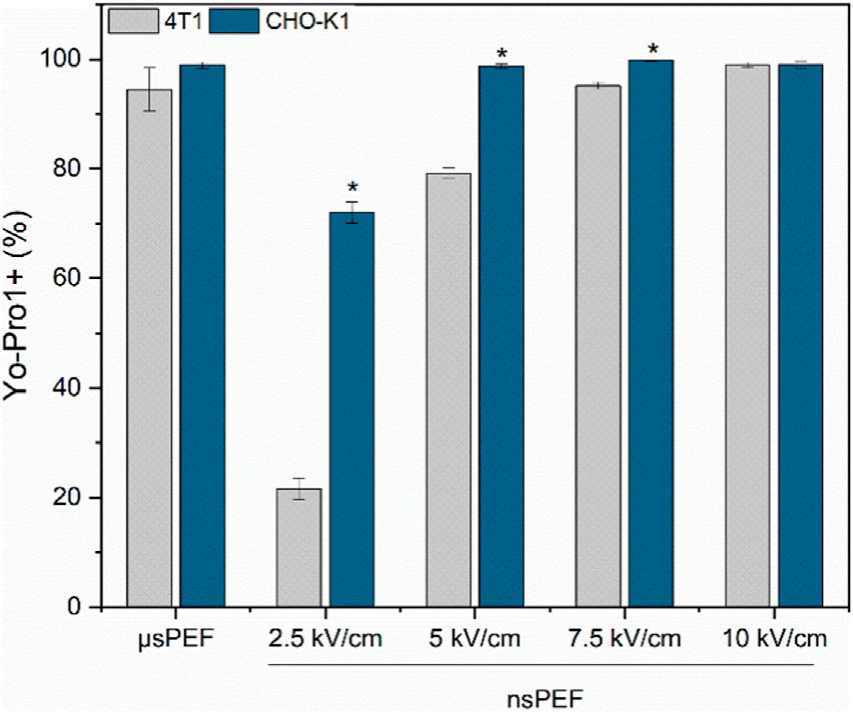
4T1 (grey) and CHO-K1 (blue) cell membrane permeabilization to Yo-Pro1 fluorescent marker. Experiments were performed using No.1 EP buffer. μs PEF—1.2 kV/cm × 100 μs × 8, 1 Hz and nsPEF—2.5–10 kV/cm × 300 ns × 250, 1 MHz. Asterisk (*) corresponds to statistically significant (*p* < 0.05) difference.

Both cell lines have been subjected to PEF and the electrotransfer of YP was evaluated. Based on data from Figure 2 it can be seen that bursts of 250 nanosecond pulses return similar results as bursts of 500 pulses, indicating the lack of rationale to use more pulses. Therefore, in order to augment the study’s versatility, we have constrained the protocols to employ 250 pulses but characterized the effects of pulse amplitude in 2.5–10 kV/cm range (standard electroporation buffer was used No. 1). The results are summarized in Figure 4.

It is apparent that the susceptibility of 4T1 cells to PEF is lower compared to the CHO-K1 cell line. For the ESOPE protocol (1.2 kV/cm x 100 µs × 8) the differences are low with both cell lines experiencing high permeabilization, although the difference is more profound in the nanosecond range. It is indicatory that the same nanosecond protocols will trigger lower GET efficiency for 4T1 cell line if the PEF parameters are not adjusted.

**FIGURE 5:**
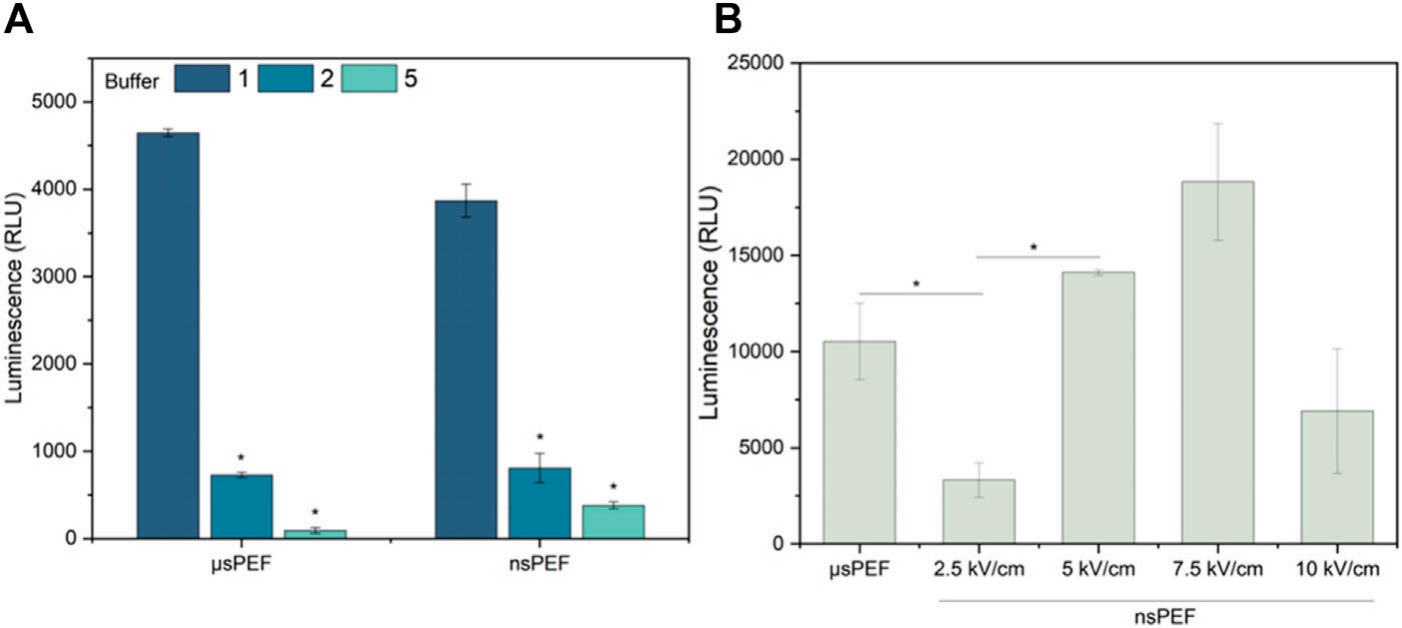
**(A)** CHO-K1 cell line electrotransfection efficiency dependence on EP buffers composition (No. 1, two and 5) with Luciferase-pcDNA3 plasmid, where µsPEF 1.2 kV/cm × 100 μs × 8, 1 Hz and nsPEF 5 kV/cm × 300 ns × 250, 1 MHz. **(B)** 4T1 cell line electrotransfection efficiency during µsPEF (1.2 kV/cm × 100 μs × 8, 1 Hz) and nsPEF (2.5–10 kV/cm × 300 ns × 250, 1 MHz), with Luciferase-pcDNA3 plasmid (EP buffer No.1). Asterisk (*) corresponds to statistically significant (*p* < 0.05) difference.

Further, the efficiency of GET with the Luciferase-pcDNA3 plasmid and three buffers (No. 1, 2, 5) was characterized using 1.2 kV/cm × 100 μs × 8, 1 Hz and nsPEF 5 kV/cm × 300 ns × 250, 1 MHz protocols and CHO-K1 cell line. The Luciferase-pcDNA3 plasmid was further utilized due to its applicability in developing a long-term transfected cell line (Figure 5A).

As can be seen (Figure 5A) the influence of buffer is more profound when another plasmid is used, thus, the results can hardly be superpositioned with standard GET characterization procedures using typical model plasmid (Figure 2). Unexpectedly, buffer No. 1 (standard electroporation buffer) is several-fold superior to buffer No. Two and five, which was not the case with GFP-encoding plasmid. Based on the dramatic results, the GET experiments with 4T1 cell line were limited to buffer No. 1, but GET with luciferase-pcDNA3 plasmid were performed in the whole range of PEF amplitudes (2.5–10 kV/cm). The results are summarized in Figure 5B.

**FIGURE 6:**
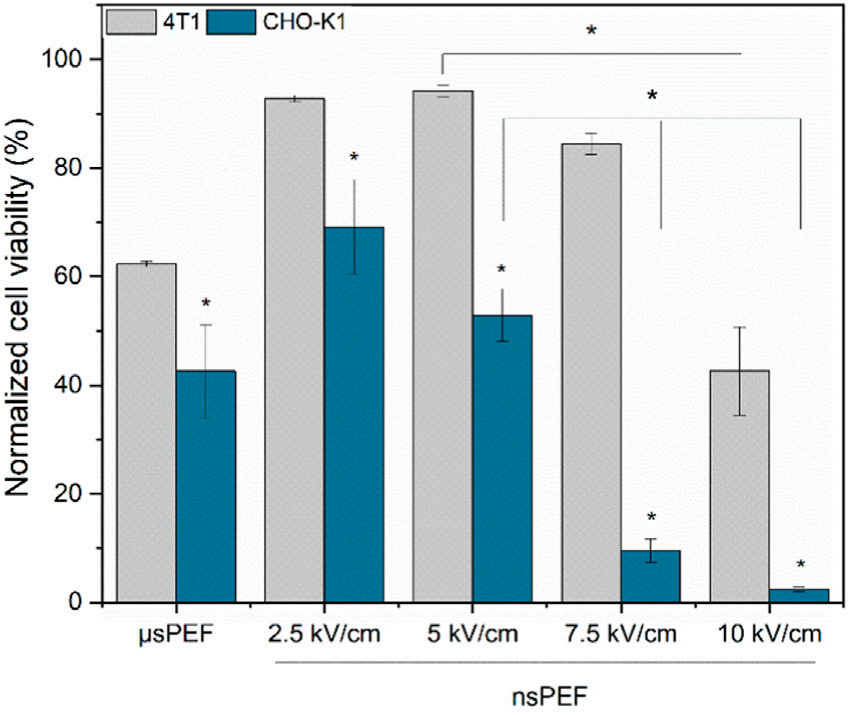
CHO-K1 (blue) and 4T1 (grey) normalized cell viability after electroporation with Luciferase-pcDNA3 plasmid. Both experiments were performed using No.1 EP buffer. Asterisk (*) corresponds to statistically significant (*p* < 0.05) difference.

As predicted, by the YP data (Figure 4) nanosecond duration (300 ns) 5 kV/cm protocol is not optimal since does not trigger saturated permeabilization. At the same time 7.5 kV/cm protocol is the best, which is in perfect agreement with permeabilization data. The 10 kV/cm protocol results in GET efficiency decline, which is attributed to significant viability loss due to irreversible electroporation. The results confirming this hypothesis are presented in Figure 6.

As it can be seen, CHO-K1 cell line is significantly more susceptible to the treatment resulting in a rapid decline of viability following the GET procedure. It’s not the case with the 4T1 cell line, which in all cases shows higher viability when compared to CHO-K1, which again proves the hypothesis that for each specific cell line protocols should be checked and adjusted to ensure high GET efficiency.

**FIGURE 7:**
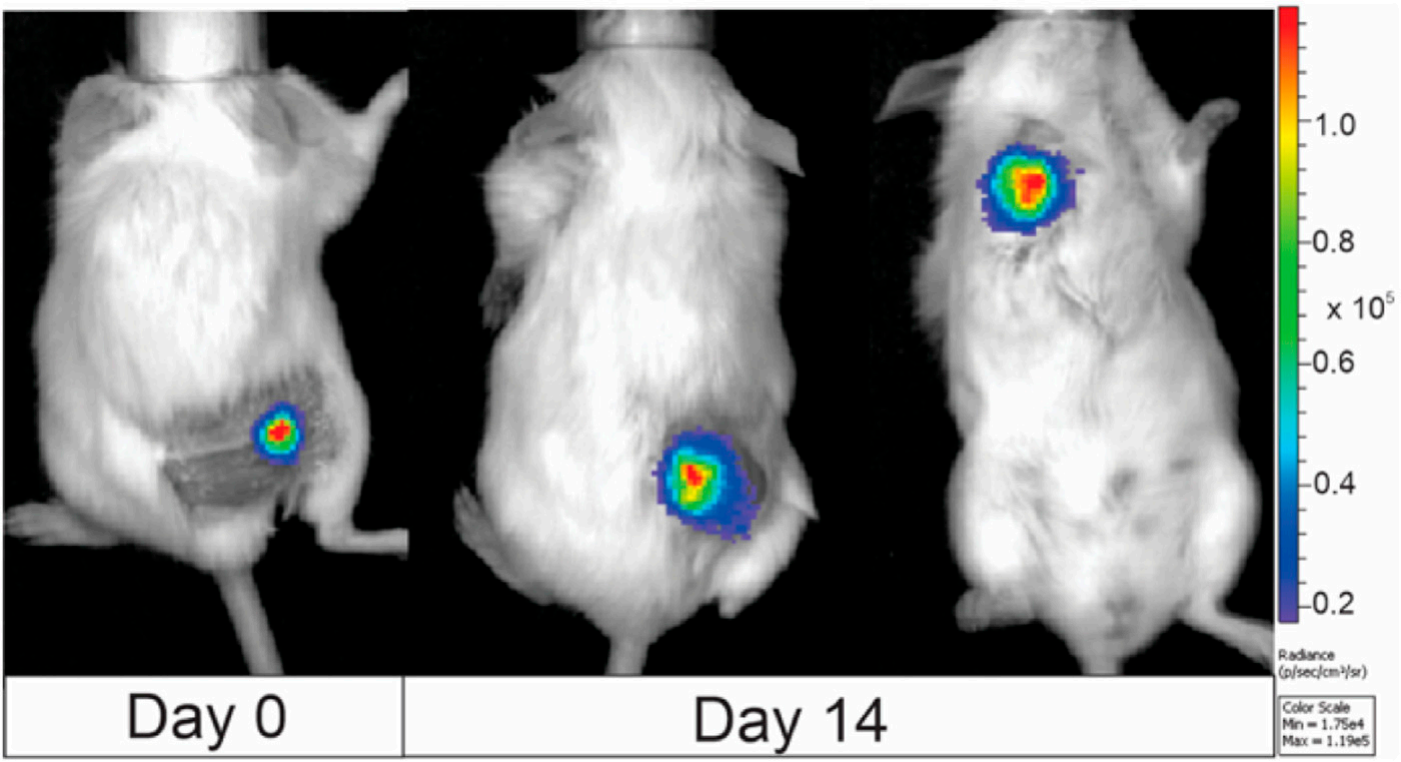
BALB/c murine imaging, where 4T1-luc clone was used for tumor induction. Images were taken at day 0 (when tumor volume reached 50 mm^3^) and day 14. The color bar on the right side of the image illustrates the correlation between color and light intensity, measured in arbitrary units (counts), for the entire animal images. Images were taken with IVIS Spectrum device and analyzed by Living Image software.

Based on the results, the 7.5 kV/cm × 300 ns × 250 pulses bursts delivered at 1 MHz using a standard electroporation buffer (No. 1) have been selected as an optimal protocol for long-term transfection to develop a luminescent clone for *in vivo* research and tumor visualization, which was a success. The exemplary images of animals featuring 4T1-Luc tumors are shown in Figure 7.

It is evidential that the developed luminescent 4T1 cell clone can be used for live tumor visualization in an *in vivo* model, and the dynamics of tumor development can be monitored (Refer to Figure 7). Also, the extent and localization of metastases (Figure 7; third mouse on the right-hand side) can be characterized both quantitatively (intensity of luminescence) and qualitatively. It also offers an excellent tool for the study of electrochemotherapy and other PEF-based techniques since it enables more precise electrode positioning and characterization of the effects after the treatment.

## 4 Discussion

The primary objective of this study was to characterize the effects of electroporation buffers on GET efficiency across both nanosecond and microsecond pulse duration ranges. Additionally, the study aimed to emphasize that using the model cell line (CHO-K1) and a standard GFP-encoding plasmid presents significant limitations when predicting GET efficiency with other plasmids or cell lines.

As already mentioned, *in vitro* GET efficiency depends on many factors, ranging from parametric electroporation conditions to the quality of the plasmid and cell line used (Golzio et al., 1998; Čegovnik and Novaković, 2004; Lesueur et al., 2016; Desai et al., 2017; Chopra et al., 2020; Sherba et al., 2020; Radzevičiūtė et al., 2022b). To evaluate transfection efficacy, we employed two distinct plasmids encoding different proteins: green fluorescent protein (pEGFP-N1) and luciferase (Luciferase-pcDNA3). The application of CHO-K1 cell line (Golzio et al., 1998; Čegovnik and Novaković, 2004; Desai et al., 2017), pEGFP-N1 plasmid (Chopinet et al., 2013; Novickij et al., 2020), and microsecond duration EP protocols was selected based on the literature as most commonly used in the *in vitro* GET context, serving as standard reference for efficiency comparison.

First, we tested the transfection efficacy of the standard CHO-K1 cell line and with pEGFP-N1 plasmid encoding green fluorescent protein (GFP) using eight different composition EP buffers and both microsecond and nanosecond range EP protocols. The highest transfection efficiency was achieved when No. 1, 2, 5, and 7 composition buffers were used. This can be attributed to the presence of divalent cations (Ca^2+^ and Mg^2+^), which are known to have a significant impact on GET efficiency (Neumann et al., 1996; Hristova et al., 1997; Haberl et al., 2013; Chopra et al., 2020). Reported electrotransfection efficacy results of GFP-encoding plasmid with the CHO-K1 cell line and standard microsecond EP protocol are in good agreement with existing knowledge (Chopinet et al., 2013; Novickij et al., 2020). Regarding sub-microsecond duration EP for gene electrotransfer, based on available knowledge, our group was the first and only one to apply high-frequency sub-microsecond protocols, and the reported data here are in agreement with our previously conducted studies (Novickij et al., 2020; Radzevičiūtė et al., 2022a). Higher transfection efficiency of the GFP protein-encoding plasmid was reached when sub-microsecond duration protocols (nsPEF) were used. Chopinet and colleagues combined classical microsecond GET protocols with nsPEFs, and their study results revealed that neither the percentage of electrotransfected cells nor the amount of GFP expressed was increased by combination with short duration nsPEF (10, 15 and 18 ns) (Chopinet et al., 2013).

Furthermore, EP buffers were narrowed down to those most effective, and we tested how they influence cell viability post-treatment, as this is another very important characteristic in GET context. Studies showed that EP No. Five buffer composition negatively affects cell viability, although it achieves sufficient electrotransfection rates. These results might be explained due to the absence of Ca^2+^ ions in the No. Five EP buffer composition, which is reported to play a crucial role (Suzuki et al., 2003; Ciobanu et al., 2018; Shi et al., 2022; Li and Shaw, 2023). Additionally, we demonstrated that the buffers containing the pEGFP-N1 plasmid are not inherently toxic to the cells. This is a favorable outcome, considering that only the plasmid’s toxicity to the cells has been previously reported (Lesueur et al., 2016). However, when combined with an PEF, the overall toxicity of the treatment increases significantly, and this increase is highly dependent on the buffer composition (Figure 3). It might be related to.

Nevertheless, to develop a luminescent murine cancer cell line for *in vivo* studies, we carried out electrotransfection protocol optimization with the Luciferase-pcDNA3 plasmid encoding luciferase protein. First, the luciferase-pcDNA3 plasmid electrotransfection efficiency of the model CHO-K1 cell line with three different composition EP buffers was tested (No. 1, 2, and 5) (Figure 4A). It should be noted that standard electroporation buffers (No. 1, 2, and 5) showed the best efficacy of transfection and competitive viability results when compared to commercial or other buffers involved in the study. Other studies showed that significantly higher transfection efficiency can be achieved when sucrose, as the osmotic balancing agent, in EP buffer is used (No.1). Our findings align with existing knowledge in the field (Sherba et al., 2020). The same electrotransfection tendencies can be observed regardless of the pulsed electric field protocol used (µsPEF or nsPEF). Furthermore, electrotransfection studies with murine 4T1 cell line were narrowed to one EP buffer composition but expanded in terms of PEFs amplitude flexibility. As seen in Figure 4B, better transfection efficiency and higher luminescence were achieved compared to standard GET protocols when applying a high-frequency nanosecond electroporation protocol with a higher amplitude (7.5 kV/cm). In the case of the highest amplitude (10 kV/cm), diminished luminescence can be attributed to potential negative effects on cell viability. Previously we reported that electrotransfection efficiency as well as cell viability is directly related to the used PEFs amplitude (Novickij et al., 2020).

However, to determine the optimal long-term transfection protocol, not only does the transfection efficacy need to be elevated, but also high permeabilization and cell viability must be ensured (Figure 5). Therefore, long-term transfection was performed utilizing nanosecond duration (7.5 kV/cm × 300 ns × 250, 1 MHz) EP protocol that resulted in a highly luminescent clone of the 4T1 cell line, which was subsequently tested in an *in vivo* murine model. As illustrated in Figure 7, even 14 days after the tumor reaches a volume of 50 mm³, the luminescence of the cancerous cells, as well as the presence of metastases, can still be detected. To the best of our knowledge, it’s the first murine cancer cell line study to confirm the successful applicability of high frequency nsPEFs in long-term electrotransfection context. The scientific data confirm the ability to long-term transfect the 4T1 mouse mammary tumor cell line with a luciferase-expressing plasmid; however, only when applying viral transfection methodology (Tao et al., 2008; Baklaushev et al., 2015; Baklaushev et al., 2017). Tao and colleagues characterized the luciferase-expressing 4T1 cell line transfected with vectors in a female BALB/c mice model by conducting a 6-week study of primary tumor growth and metastasis. As in the case of our study, the transfected 4T1 cell line allowed for the assessment of tumor growth and metastasis progression (Tao et al., 2008). Additionally, we have previously proved that high-frequency nanosecond duration EP protocols can be used for long-term transfection, ensuring significantly higher luminescence compared to standard GET protocol (μsPEF). However, our previous study focused on CHO-K1 cell line, which is the most commonly utilized platform for recombinant protein expression (Radzevičiūtė et al., 2022b), while most of the time transfection of cancerous cell lines is more challenging due to genetic instability. Thus, we demonstrate an efficient electrotransfection methodology for mammalian cells using high-frequency pulsed electric fields (PEFs), which represents a significant enhancement over standard existing GET methodologies.

## 5 Conclusion

In summary it has been demonstrated that the properties of the electroporation buffer, such as ionic composition, used during EP treatment, have a significant impact on gene electrotransfer efficiency. Additionally, we showed that sub-microsecond duration protocols reached higher, or on-par transfection efficiency and cell viability when compared to standard microsecond modality. As a proof of concept, we established long-term transfected luminescent cell line using high-frequency nanosecond EP. Also, the developed murine 4T1-Luc cell line successfully validated its suitability for utilization in an *in vivo* model for tumor and metastasis evaluation.

## Data Availability

The original contributions presented in the study are included in the article/Supplementary Material, further inquiries can be directed to the corresponding authors.
